# A recombinant scFv-FasL_ext_ as a targeting cytotoxic agent against human Jurkat-Ras cancer

**DOI:** 10.1186/1423-0127-20-16

**Published:** 2013-03-05

**Authors:** Derek V Chan, Rahul Sharma, Chiao-Ying A Ju, Steve R Roffler, Shyr-Te Ju

**Affiliations:** 1 Department of Pathology, Boston University School of Medicine, Boston University, Boston, MA 02118, USA; 2 Center for Immunity, Inflammation, and Regenerative Medicine, Department of Medicine, University of Virginia, Charlottesville, VA 22908, USA; 3 Institute of Biomedical Sciences, Academia Sinica, Taipei, Taiwan; 4 Current address: Department of Dermatology, Ohio State University Medical Center, Columbus, OH 43221, USA

## Abstract

**Background:**

Targeted therapy of human cancers is an attractive approach and has been investigated with limited success. We have developed novel cytotoxic agents for targeted therapy of human cancers based on the extracellular cytotoxicity domain of CD178 (FasL) and the specificity offered by single chain antibodies (scFv) against dominant human tumor Ag TAG-72 (cc49scFv) and TAL6 (L6scFv).

**Results:**

The cc49scFv-FasL_ext_ is highly effective in *in vitro* killing of human TAG-72^+^ Jurkat-Ras tumor cells with a 30,000 fold greater cytotoxicity as compared to soluble FasL (sFasL). On the other hand, L6scFv-FasL_ext_ only increased cytotoxicity 500-fold as compared with sFasL against TAL6^+^ HeLa cells in *in vitro* assays. The high specificity and strong cytotoxicity of cc49scFv-FasL_ext_ made it feasible to cure IP-implanted Jurkat-Ras tumors in SCID mice.

**Conclusion:**

Our study demonstrated that scFv-FasL_ext_ with a strong cytotoxicity against sensitive human tumor targets may be useful as effective chemotherapeutic agents.

## Background

Targeted therapy of human cancers has been considered by many investigators and remains an attractive approach to avoid side effects of cancer treatment. Anti-CD95 (Fas) Ab that induces Fas^+^ tumor apoptosis *in vitro* has been investigated as a potential reagent for this purpose [1]. One approach to increase the specificity of the killing is to link the FasL extracellular cytotoxic domain (FasL_ext_) with a tumor Ag specific single chain Ab (scFv). Such fusion proteins may attract the binding of FasL_ext_ only to tumor cells and selectively induce apoptosis of cancer cells.

A study based on similar concept had been reported in a mouse tumor model [2]. An scFv derived from anti-fibroblast activation protein (FAP) Ab was fused with FasL_ext_. It was clearly demonstrated that such a fusion protein did not induce liver toxicity [2]. Interestingly, local treatment with the fusion protein against Fas^+^ cancer cells that had been transfected with FAP prevented tumor growth in the skin whereas un-transfected tumor cells were marginally inhibited [2]. Apparently, the FAP-transfected cancer cells continuously provided the source that allowed aggregate formation of the fusion protein such that strong cytotoxicity against the Fas^+^ cancer cells could be induced [2]. In another related study, it was demonstrated that CTLA-4-FasL_ext_ recombinant protein did not display liver toxicity. It also did not express cytotoxicity against Fas^+^ target cells *in vitro* unless B7-bearing cells were added. Apparently, only through forming FasL aggregate on the B7^+^ cells could the toxicity be induced [3].

In contrast to the above studies, we generated two recombinant scFv-FasL_ext_ fusion proteins using scFv derived from cc49 and anti-TAL6 mAb, which target human tumor-associated glycoprotein (TAG-72) and tumor-associated Ag L6 (TAL6), respectively [4,5]. As compared with soluble FasL (sFasL), L6scFv-FasL_ext_ was 500-fold more cytotoxic against TAL6^+^ HeLa cells and cc49scFv-FasL_ext_ was 30,000-fold more cytotoxic against TAG-72^+^ Jurkat lymphoma cells. The cc49- and L6- scFv-FasL_ext_ fusion proteins serve as important reciprocal controls, demonstrating the specificity and sensitivity of cytotoxicity against Jurkat and HeLa cells respectively *in vitro*. In animal models, cc49scFv-FasL_ext_ cured Jurkat-Ras (Jurkat cells transfected with human Ras oncogene) tumors implanted in SCID mice owing to increased sensitivity to the recombinant protein depending on TAG-72 and Fas expression as well as high-sensitivity of the tumor to cc49scFv-FasL_ext_. Our study raises the possibility that certain human cancers may be highly sensitive to cc49scFv-FasL_ext_ such that they may be treated with the bioactive reagent.

## Methods

### Animals, cancer cells, and immunochemical reagents

The immune compromised SCID mice on BALB/c background (CBySmn.CB17-*Prkdc*^*scid*^) and *Rag1*^*-/-*^ mice in B6 background (B6.129S7-*Rag1*^*tm1Mon*^/j) were obtained from the Jackson Laboratories, Bar Harbor, Maine, USA. Anti-TAG-72 (cc83) and anti-L6 mAb were provided by co-author Dr. S. Roffler. All human cancer cells with the exception of Jurkat-Ras were obtained either from ATCC or the tissue culture center of the University of Virginia. The Jurkat-Ras cell line was kindly provided by Dr. D. Faller at the Boston University School of Medicine. Experiments involving animals were conducted in accordance with the protocols approved by the Animal Care and Use Committee (ACUC) of the University of Virginia.

### Plasmids

The plasmid p2c11-γ1-B7, containing 2c11scFv encoding anti-mouse CD3ε chain, pLHCX-cc49-hβG, a construct encoding cc49scFv, and pET22b-L6, a construct encoding L6scFv, were prepared by Liao et al and Chou et al [6,7]. pBluescript II KS-hFasL, a plasmid containing the human FasL, was a gift from Dr. S. Nagata [8].

### Construction of cc49scFv-FasL_ext_ and L6scFv-FasL_ext_

The scFv-FasL_ext_ constructs were first assembled in a pHook-based vector. This involved first making 2c11scFv-FasL_ext_ from p2c11scFv-γ1-B7 construct by replacing the γ1-B7 portion with FasL_ext_. Subsequently, 2c11scFv was replaced with cc49scFv and L6scFv.

To generate the pHook 2c11scFv-FasL_ext_ fusion protein construct, the γ1-B7 portion of p2c11-γ1-B7 was replaced with FasL_ext_. The following primers were used to amplify FasL_ext_ from pBluescript II KS-hFasL: 5^′^- CGGACCAGGACAAGTACAACTACAAGAAAAAAAGGAGCTGAGG -3^′^ and 5^′^- TCTAGAATTCTCGAGTTAGAGCTTATATAAGCCGAAAAACGTCTG -3^′^ using Hotstart PFU Turbo (Stratagene), according to the manufacturer’s directions. Subsequently, T4 polynucleotide kinase (New England Biolabs) was used to blunt end the PCR product. Afterward, Xho I was used to digest the PCR product and the PCR mixture was electrophoresed. A band of about 460 bp was excised from the gel and purified using a QIAEX II gel extraction kit (Qiagen). In a separate reaction, pHook-scFv-γ1-B7 was digested with Sal I, filled in with Polymerase Klenow Fragment, cut with Xho I, and ligated with the FasL_ext_ fragment.

To generate cc49scFv-FasL_ext_ and L6scFv-FasL_ext_, the 2c11scFv fragment was excised from pHook 2c11scFv-FasL_ext_ using Sfi I and Sal I restriction enzymes, and replaced with the PCR product coding for either the cc49scFv or L6scFv digested with Sfi I and Sal I. The primers used to amplify cc49scFv, L6scFv, and incorporate the Sfi I and Sal I restriction sites at either end were; cc49FP 5^′^-TGCCATGGCCCAGCCGGCCCAGGTTCAGTTGCAGCAGTCTGA-3^′^ and cc49RP 5^′^- TCCTGGTCCGTCGACTCCGCTTCCTCCGCTTCCTTTCAGCACCAGCTTGGTCCCAG-3^′^; L6FP: 5^′^- ATAGGTTGCCATGGCCCAGCCGGCCATTGTTCTCTCCCAGTCTCCAGCA -3^′^ and L6RP 5^′^- TCCTGGTCCGTCGACTCCGCTTCCTCCGCTTCCGGATGAGGAGACTGTGAGAGTGGT-3^′^.

To obtain higher expression levels, the cc49scFv-FasL_ext_ and L6scFv-FasL_ext_ expression cassettes were PCR amplified from the pHook based vectors using the following common primers, digested with Xho I and Not I restriction enzymes and cloned in the pBCMGSNeo vector at the Xho I and Not I sites. BCMGXhoI FP 5^′^- ATCGCCT CTAATCTCGAGATGGAGACAGACACACTCCTGCTA-3^′^ and BCMGNotI RP 5^′^- GGCCTCGACTCGCA TGCGGCCGCTTAGAGCTTATATAAGCC GAA-3^′^.

### Generation of cell lines expressing the fusion proteins

Transfection of plasmids was performed using lipofectamine (Invitrogen) or electroporation at 960 μF capacitance and voltages of 270 mV, 280 mV, or 290 mV using a BioRad Gene Pulser (BioRad). Ten million NIH-3T3 or BW5147 cells were utilized for each transfection. Immediately after transfection, cells were grown in DMEM (Invitrogen) containing 10% heat-inactivated FCS, vitamin solution (Cellgro), 100 mM sodium pyruvate solution, and penicillin-streptomycin-glutamine (Invitrogen). Two days later, 0.6 mg/ml of G418 (Invitrogen) was added to cultures and cells were allowed to adapt over a 2-week period. Cells were then transferred to fresh media containing 1 mg/ml of G418. Cell cultures were split as necessary, while maintaining G418 concentrations. After the mock transfected cells were dead, the mixtures were cultured for an additional 2 weeks in media containing G418. Subsequently, culture supernatants were collected and screened for cytotoxic activity against A20 cells. Cell populations whose supernatant expressed cytotoxicity were expanded for subsequent use in assays. Cells from wells with strongly cytotoxic supernatants were adapted to normal culture medium and expanded for further studies.

### Flow cytometric analysis

Half a million cells were stained for Fas expression using PE-DX2 anti-Fas (Pharmingen) mAb, and with PE-mouse isotype control (Pharmingen). Expression analysis of L6 and TAG-72 was done using a two-step staining. L6 mAb, IgG2a isotype control (G155-178, Pharmingen), cc83 ascites fluid (a second generation mAb made against purified TAG-72) [5], and heat-inactivated BALB/c serum were used first, followed by washing and staining with fluorescent secondary Ab. For two-step staining using scFv-FasL_ext_, 1 ml of respective transfected cell culture supernatant was added to each cell pellet. Cells were incubated on ice in the dark for 45 minutes. Cells were rinsed 2 times in PBS and supernatants were removed. Cells were then stained with FITC- or PE-goat anti-mouse IgG (Southern Biotech), PE-Alf 1.2 anti-FasL (Caltag), and PE-mouse IgG2a isotype control (Caltag) secondary Ab. Cells were incubated on ice in the dark for 45 minutes, rinsed 2 times with PBS, and then fixed in 2% paraformaldehyde. Samples were analyzed using CellQuest and a Becton-Dickenson flow cytometry machine.

### Immunoblot

40 μl of cell supernatants or media were loaded onto a 15% polyacrylamide gel using Laemmli buffer. A FasL recombinant protein corresponding to the human FasL_ext_ was used as a positive control (Santa Cruz Biotechnology) (data not shown). Proteins were resolved at 40 mA and transferred at 150 V for 75 minutes onto PVDF membranes using a BioRad MiniProtean II system (BioRad).

Membranes were then blotted with rabbit polyclonal anti-HA or rabbit polyclonal anti-FasL (C-20) (Santa Cruz Biotechnology) followed by HRP-conjugated donkey anti-rabbit Ab [HRP] (Amersham Biosciences). Bound HRP were exposed to ECL reagents (Amersham Biosciences) and results were recorded using X-OMAT AR film (Kodak).

### Size exclusion analysis

Culture supernatants were fractionated using a Millipore molecular membrane filtration apparatus that separates molecules based on their sizes above or below 300 kDa. The volumes of both the “Flow-through” and “Retentate” fractions were brought to original volume with fresh culture medium and examined for cytotoxicity against A20 target cells.

### Quantification of FasL

The scFv-FasL_ext_ concentration in supernatants was determined using a commercial kit for sFasL (Oncogene Science). The molecular mass of sFasL was assumed to be 26 kDa based on gene sequence data. The molecular mass of the fusion proteins was estimated to be 54 kDa. A standard curve was generated. Absorbance values of samples that fell within the linear range of the standard curve were used to determine the original sample concentrations.

### Cytotoxicity assays

Cytotoxicity assays against various target cells were conducted as previously described. Target cells (2×10^4^), labeled with Na_2_^51^CrO_4_ (New England Nuclear), were cultured with various doses of Jo2 mAb (BD Pharmingen) or culture supernatant in 0.2 ml in individual wells of a 96-well plate for 5 hours in a 37°C, 10% CO_2_ incubator [9,10]. Supernatants were removed and radioactivity (cpm) was determined using an LKB-Wallac gamma counter (Perkin-Elmer). Background release was determined by culturing cells alone. Cytotoxicity is expressed as% specific ^51^Cr release, which is determined by the formula: 100*%* × (experimental release–background release)/(total cpm released by 0.5*%* NP − 40–background release). Assays were carried out in duplicate and the experiment was repeated three times. For adherent human cancer cell lines such as HeLa, LS-174 T, and several breast cancer cell lines, cells in 96-well plate were either treated overnight with IFN-γ (100 ng/ml) or left untreated followed by the addition of the cytotoxic proteins. The standard MTT (3-(4,5-Dimethylthiazol-2-yl)-2,5-diphenyltetrazolium bromide) (Sigma) assays were conducted 18 hours after IFN-γ treatment [11]. In some experiments, cytotoxicity assays were conducted in the presence of inhibitors such as Alf 1.2 anti-FasL, NOK-1 anti-FasL [12], cc83 anti-TAG-72 [5] or anti-L6 [6] mAb with isotype included as appropriate controls.

### Treatment of cancer cells *in vivo*

We could not grow Jurkat cells in SCID and *Rag1*^*-/-*^ mice using (30-50) ×10^6^ cells per IP injection even at 4 months after implantation. However, tumor growth was readily achieved by using Jurkat-Ras cells. This dose was used for the treatment studies with cc49scFv-FasL_ext_. SCID mice implanted IP with Jurkat-Ras cells and not treated with the cc49scFv-FasL_ext_ fusion protein usually died within 5 weeks.

Two days after tumor implantation, scFv-FasL_ext_ containing supernatant (1 ml for cc49scFv-FasL_ext_ containing approximately 2 μg FasL equivalent, based on ELISA assay) was injected IP into the peritoneum every other day for as long as 4 weeks or until the mice died. In some experiments, the first injection of cc49scFv-FasL_ext_ was administered at 6 days after tumor implantation.

## Results

### Derivation and immunological characterization of scFv-FasL_ext_

**Figure 1:**
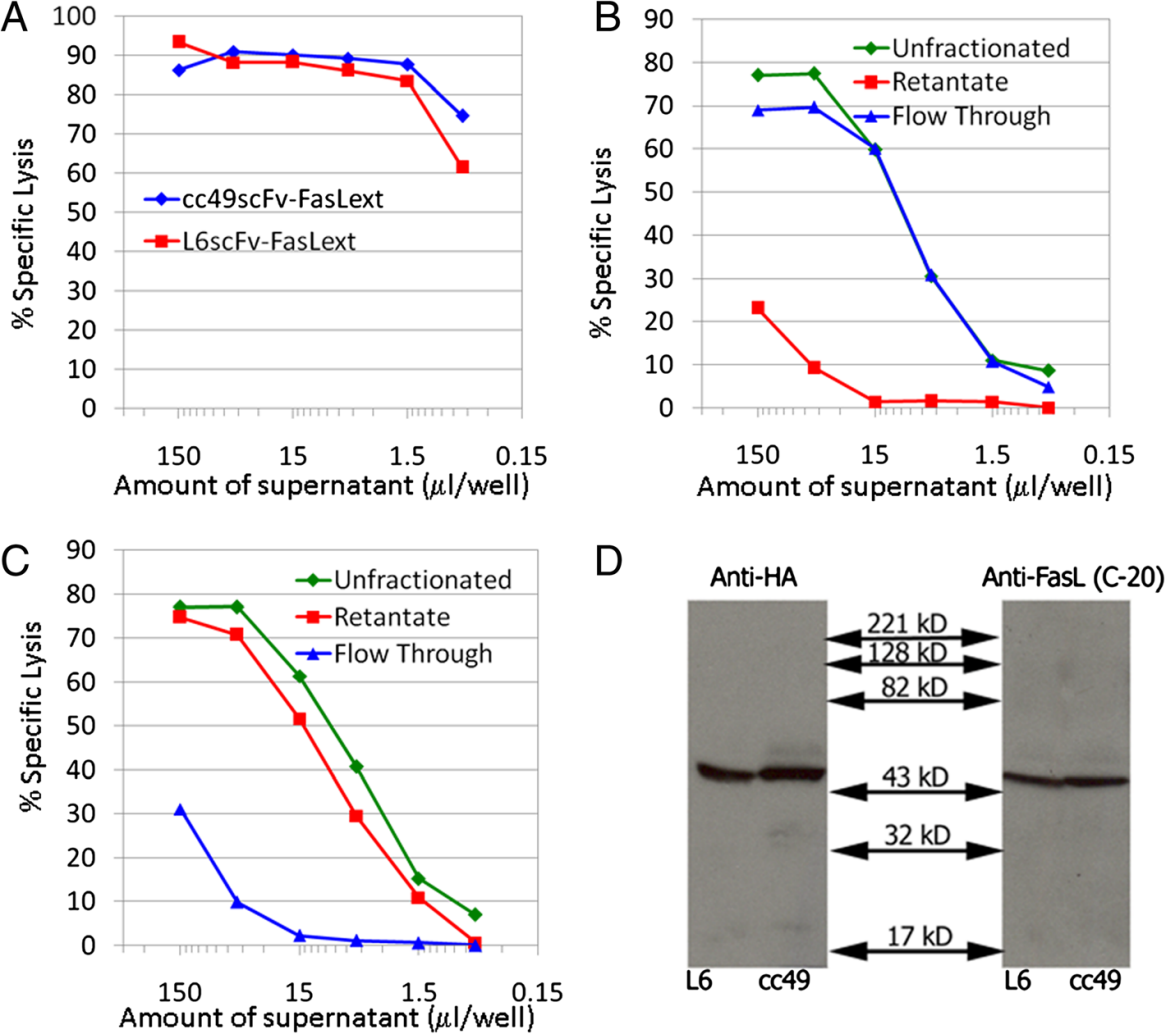
**Immunochemical characterization of cc49scFv-FasL**_**ext **_**and L6scFv-FasL**_**ext**_**.** (**A**) Cytotoxicity was detected against A20 target by using culture supernatants from cc49scFv-FasL_ext_ and L6scFv-FasL_ext_ transfected BW5147 and 3T3, respectively. Culture supernatants from BW5147 and 3T3 had no detectable cytotoxicity against A20. (**B** and **C**) Membrane filtration analysis of cytotoxic materials: A 300 kDa membrane molecular filtration was used as described in Methods. Flow-through and “Retentate” of cc49scFv-FasL_ext_ (**B**) and L6scFv-FasL_ext_ (**C**) samples were readjusted to original volume and tested for cytotoxicity against A20. (**D**) Polyclonal rabbit anti-FasL (C-20) and rabbit anti-HA were used to probe for the presence of fusion proteins. Dilutions used were 1000 fold for anti-HA, 1000 fold for anti-C-20, and 4000 fold for horseradish peroxidase-conjugated anti-rabbit Ab.

We transfected several cell lines with the recombinant cc49scFv-FasL_ext_ and L6scFv-FasL_ext_ constructs and used limiting dilution technique to obtain the clones that produced the highest cytotoxicity against the A20 tumor cells, which express Fas Ag and are sensitive to sFasL-mediated cytotoxicity [12]. The selected clones were then gradually adapted to growth in normal culture medium without the selection agent. As shown in Figure 1A, culture supernatants for two clones, cc49scFv-FasL_ext_ and L6scFv-FasL_ext_ transfectants, but not the supernatants from BW5147 and 3T3 (see legend), were shown to have strong cytotoxicity against the A20 target cells. In addition, culture supernatant of these clones adapted to grow in serum-free media was also strongly cytotoxic (data not shown). The results showed that the cytotoxic components were derived from the transfected recombinant genes, i.e., scFv-FasL_ext_.

### Biochemical characterization of scFv-FasL_ext_

We determined the molecular sizes of the cytotoxic components secreted by the scFv-FasL_ext_ transfected cells by molecular membrane filtration through a 300 kDa Millipore filter. As shown in Figure 1B, the large proportion of Fas-dependent cytotoxicity was present in the “Retentate” of the L6scFv-FasL_ext_ whereas the great majority of cytotoxicity was present in the “Flow-through” of cc49scFv-FasL_ext_ supernatant (Figure 1C). The result indicated that individual scFv-FasL_ext_ form different levels of multimeric structures. The cytotoxic activities in the respective fractions were verified by Western blotting to correspond to the scFv-FasLext fusion proteins (not shown). Western blotting analyses of the culture supernatants from both clones using polyclonal anti-HA for scFv and anti-C20 FasL_ext_ Ab demonstrated that the scFv-FasL_ext_ fusion proteins expressed the expected molecular size under denaturing and reducing conditions (Figure 1D).

### Targeting cancer Ag specifically increases cytotoxicity against the Ag-bearing human cancer cells

**Figure 2:**
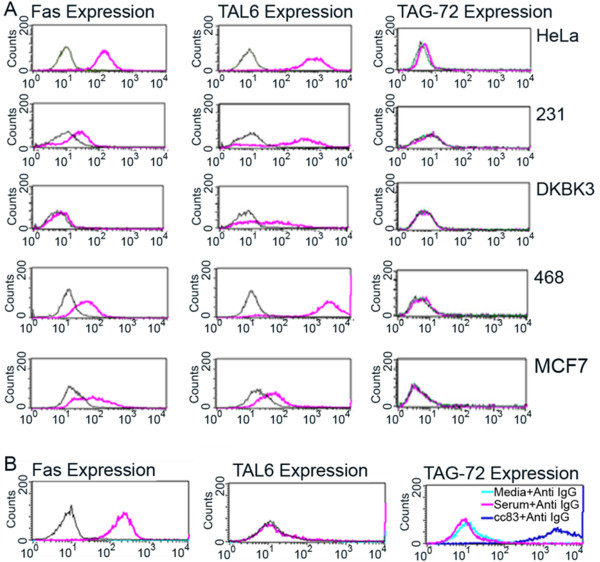
**Expression of cancer Ag and Fas on various human cancers.** (**A**) HeLa, 231, DKBK3, 468, and MCF7 cell lines were analyzed by flow cytometry for Fas, TAL6, and TAG-72 expression: left panels (Fas expression); center panels (TAL6 expression); right panels (TAG-72 expression). All positive stains were indicated by red lines. Background staining was indicated by green or gray lines. (**B**) Expression of Fas (red line-left panel), TAL6 (red line-middle panel) and TAG-72 (dark blue-right panel) on Jurkat cells. Jurkat cells are Fas^+^TAL6^-^TAG-72^+^.

**Figure 3:**
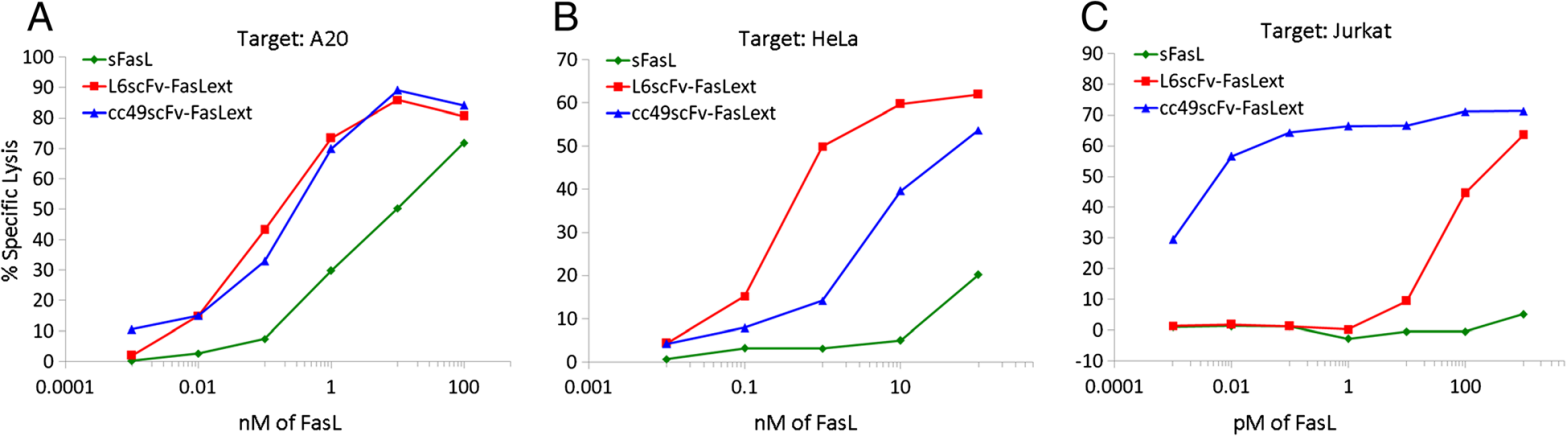
**Increase of sensitivity of human cancer target cells bearing TAG-72 or TAL6 toward cc49-FasL**_**ext **_**and L6scFv-FasL**_**ext**_**, respectively.** The cytotoxicity against A-20 and Jurkat targets were conducted with 5-hour ^51^Cr release assays. The cytotoxicity against HeLa targets was conducted in 24-hour MTT assays. Please note that x-axis scale of panel **C** is on a pM scale.

We chose a positive way to demonstrate the strong association between TAG-72 and Fas for the increase in cytotoxicity of cc49scFv-FasL_ext_. TAL6 and TAG-72 that are broadly expressed on a variety of human cancer cell lines were examined for their potential as appropriate targets for scFv-FasL_ext_. The expression of Fas, TAL6, and TAG-72 on tumor cells was determined by Flow cytometry (Figure 2). Among a half dozen human cancer cells, significant expression of these Ag are observed although with great variability. Among these cancer cell lines, HeLa (TAL6^+^Fas^+^) was the most sensitive target to L6scFv-FasL_ext_ and Jurkat (TAG-72^+^Fas^+^) was the most sensitive one to cc49-FasL_ext_ (Figure 3 below). Human cancer line LS-174T also expressed both Fas and TAG-72 [5] but they were much less sensitive than Jurkat to cc49-FasL_ext_ (data not shown). In all cases, we noticed that increased sensitivity toward scFv-FasL_ext_ required the strong expression of both tumor Ag and Fas.

In another series of experiments, we included A20 as a control for scFv-FasL_ext_ for Fas-mediated cytotoxicity without the tumor Ag influence because mouse A20 expresses only Fas but not the human TAG-72 nor TAL6. The culture supernatants from L6scFv-FasL_ext_ and cc49scFv-FasL_ext_ showed comparable cytotoxicity against the A20 target (Figure 3A). In addition, both scFv-FasL_ext_ were more cytotoxic to A20 than sFasL. HeLa cells (TAL6^+^TAG72^-^Fas^+^) were found to be 500-fold more sensitive to L6scFv-FasL_ext_ than sFasL (Figure 3B) whereas Jurkat cells (TAL6^-^TAG-72^+^Fas^+^) were 30,000 times more sensitive to cc49scFv-FasL_ext_ than sFasL (Figure 3C). In addition, L6scFv-FasLext was significantly more cytotoxic against HeLa cells as compared to cc49scFv-FasL_ext_ (Figure 3B). Conversly, cc49scFv-FasLext displayed far more enhanced cytotoxicity against Jurkat cells as compared to L6scFv-FasL_ext_ (Figure 3C). The results clearly demonstrated that the increase in Fas-mediated cytotoxicity of scFv-FasL_ext_ required targets bearing the cognate tumor Ag.

**Figure 4:**
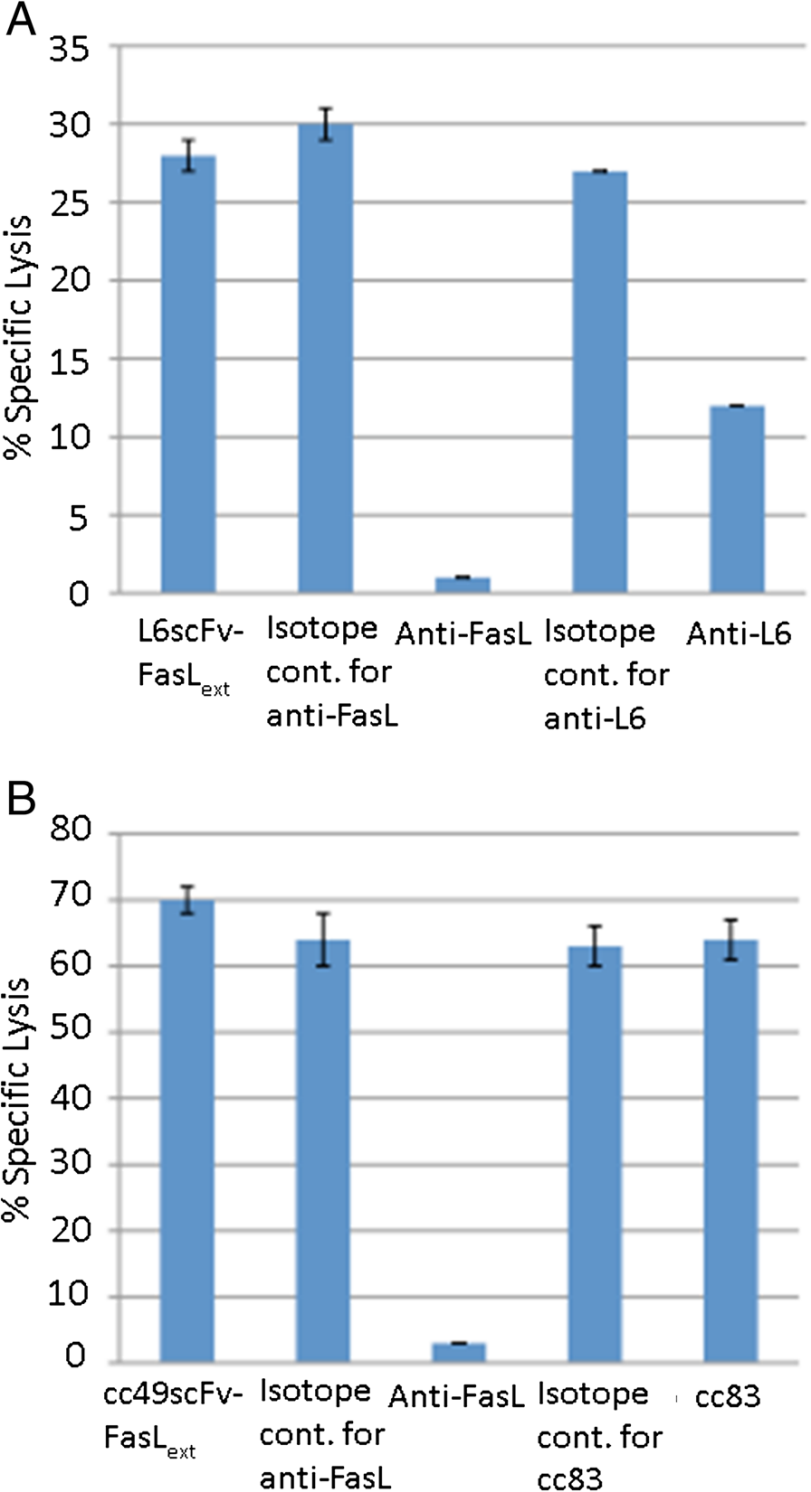
**Inhibition of cytotoxicity of fusion proteins by anti-FasL, anti-TAG-72, and anti-L6 mAbs.** Cytotoxicity against HeLa cells and cytotoxicity against Jurkat cells were shown in 2A and 2B, respectively. NOK-1 (2 μg/ml) and Alf 1.2 (2 μg/ml) anti-FasL were used as inhibitors against cc49scFv-FasL_ext_ (0.8 nM) and L6scFv-FasL_ext_ (3.2 nM), respectively. Both strongly inhibited the cytotoxicity. In contrast, cc83 (5 μg/ml) failed to inhibit cytotoxicity against Jurkat cells whereas anti-L6 (5 μg/ml) only inhibited 30% of the cytotoxicity against HeLa cells caused by their respective fusion proteins.

The tumor and Fas Ag that react with the fusion proteins was determined by inhibition of cytotoxicity. As shown in Figure 4, anti-human FasL mAb (NOK-1 or Alf 1.2) strongly inhibited the killing of HeLa cells by L6scFv-FasL_ext_ and of Jurkat cells by cc49scFv-FasL_ext_, respectively. Although the ability of anti-L6 mAb to inhibit HeLa killing by L6scFv-FasL_ext_ was moderate, the ability of cc83 to inhibit the killing of Jurkat by cc49scFv-FasL_ext_ was minimal. It is important to note that inhibition of cytotoxicity by anti-FAP and anti-CTLA-4 was not reported [2,3]. This condition may be caused by the increased binding sites against tumor Ag relative to FasL_ext_ on the recombinant proteins. The cc49scFv-FasL_ext_ also had a 3-fold increase in tumor Ag binding sites due to the ability of FasL_ext_ to form trimers. In addition, the extremely strong cytotoxicity suggests a strong expression of tumor Ag such that inhibition of cytotoxicity was difficult to achieve. Other possibilities uniquely associated with Jurkat cells are addressed in the “Discussion”.

### Treatment to increase sensitivity of TAL6^+^Fas^+^ cells to L6scFv-FasL_ext_

Because HeLa cells only showed a 500-fold increase in sensitivity toward L6scFv-FasL_ext_ relative to sFasL, we tried increasing the sensitivity of L6scFv-FasLext against TAL6^+^Fas^+^ target cells. IFN-γ has been shown to increase the sensitivity of cancer cells to Fas-mediated apoptosis [13,14]. To see if treatment with IFN-γ *in vitro* would increase their sensitivity the TAL6^+^Fas^+^ target cell lines were treated with IFN-γ as described in “Methods”. Although, the result showed that such treatment increased tumor cell sensitivity by 3-5 folds toward L6scFv-FasL_ext_ (Table 1), HeLa remained the most sensitive cancer cells examined. In *in vivo* experiments L6scFv-FasL_ext_ culture supernatant administered at various doses was unable to protect SCID mice implanted with HeLa tumors due to insufficient increase in cytotoxicity and therefore, was not studied further (data not shown).

**Table 1:** **IFN-**γ **treatment enhances the sensitivity of cancer cells to L6scFv- FasL**_**ext **_**mediated apoptosis**

**Target cells**	**% Specific killing of 10**^**4 **^**cells/well by L6scFv-FasL**_**ext **_**(μl/well)**					
	**30 μl**	**10 μl**	**3 μl**	**1 μl**	**0.3 μl**	**0.1 μl**
HeLa	84	80	64	32	4	3
IFN-γ-HeLa	87	85	62	60	46	24
468	58	53	41	11	−5	−3
IFN-γ-468	83	81	79	69	35	17
DKBK3	21	13	11	13	10	11
IFN-γ-DKBK3	78	66	44	31	17	7

Breast cancer cells were sensitized by IFN-γ *in vitro*. Under the cytotoxicity assay condition, IFN-γ (100 ng/ml) enhanced 3-10 folds of the target susceptibility to L6scFv-FasL_ext_.

### Treatment of TAG-72^+^Fas^+^ human cancer cells in SCID mice with scFv-FasL_ext_

We established tumor models in SCID mice to determine whether cc49scFv-FasL_ext_ fusion protein, which expresses extremely high cytotoxicity against Jurkat cells, is therapeutic. Jurkat cells do not form tumors in SCID mice or in *Rag1*^*-/-*^ mice transferred with as many as 50×10^6^ cells IP. However, the Jurkat-Ras cells were able to form tumors in SCID mice. The *in vitro* cytotoxicity of Jurkat-Ras did not differ from Jurkat (data not shown). We transferred 40×10^6^-50×10^6^ Jurkat-Ras cells in SCID mice, resulting in tumor growth and death at 4-5 weeks after transfer. At 2-6 days after cancer cell transfer, we started treatment by IP injection with 1 ml of cc49scFv-FasL_ext_-containing culture supernatant every other day. Control groups were injected with culture medium of BW5147 cells. Remarkably, treatment of Jurkat-Ras tumor transplants with cc49scFv-FasL_ext_-containing culture supernatants protected the mice from mortality (Table 2). The great majority of the recipients remained alive and healthy at 1-2 months after treatment whereas tumor growth was evident in control groups at this time. Tumor growth was confined in the peritoneum with individual colonies, some of which were attached to the outer surface of liver, spleen, stomach, and intestinal walls. The treated mice were free of any visible tumor cells. They remained healthy for another two months after the treatment was stopped. Eradication was accomplished since these mice remained healthy for an additional 14 weeks (Table 2). The data demonstrate a successful eradication of Jurkat-Ras tumor cells transplanted in SCID mice by targeted therapy using cc49scFv-FasL_ext_ fusion protein.

**Table 2:** **Highly cytotoxic cc49scFv-FasL**_**ext **_**eradicated Jurkat-Ras cancer in SCID mice**^**a**^

**Cells injected on day 0**	**Treatment begins on**	**# mice treated**	**Tumor incidence**	**# tumor-free mice at the end of the treatment**^**b**^
(40-50)×10^6^ Jk-Ras Control	Day 1-2	7	6 at 4-6 wk later	1
(40-50)×10^6^ Jk-Ras Test	Day 1-2	8	0 at 4-6 wk later	8
20×10^6^ Jk-Ras Control	Day 6	4	4 at 4-6 wk later	0
20×10^6^ Jk-Ras Test	Day 6	5	1 at 4-6 wk later	4

^a^SCID mice (>14 weeks old) of both sexes were transferred by IP injection of Jurkat-Ras (Jk-Ras) or HeLa at day 0. Treatment started at indicated times by IP injection of cc49scFv-FasL_ext_-containing supernatants (2 μg/ml) or concentrated L6scFv-FasL_ext_ (5-10 μg/ml) as described in the “Methods”.

^b^Two months after the end of the treatment, the survived mice still lived without any indication of toxicity induced by the treatment. Normal liver appearance was observed when survived mice were euthanized later.

## Discussion

The present study suggests that using scFv-FasL_ext_ fusion protein as a therapeutic agent against human cancers is feasible only if the target tumor is extremely sensitive to the scFv-FasL_ext_ –mediated cytotoxicity, such as the case for cc49scFv-FasLext mediated cytotoxicity against Jurkat cells. However, other than Jurkat-Ras, we have not found a second commonly used human cancer cell line that displays such sensitivity to cc49scFv-FasL_ext_ and can be cured in an animal model. In the vast repertoire of tumor diversity in human cancers, highly sensitive tumors like Jurkat cells may exist and as such may be sensitive to cc49scFv-FasL_ext_. In this respect, various cancer cells present in repositories that express Fas and TAG-72 should be examined for their sensitivity to cc49scFv-FasL_ext_. Even if a small fraction of the TAG-72^+^ human tumors were found to be nearly as sensitive as Jurkat, it would be significant that the cc49scFv-FasL_ext_ could completely eradicate the cancer cells. Moreover, our study also raises the possibility in using scFv-FasL_ext_ in combination therapy in which tumor cell sensitivity to scFv-FasL_ext_ can be enhanced by commonly used cancer treatment protocols.

Intuitively, it is difficult to understand how such a dramatic increase in cytotoxicity against Jurkat cells was observed when cc49scFv was fused with FasL_ext_. A 30,000-fold increase in cytotoxicity is not easily comprehended simply by adding a co-operative ligand to aid for binding. Several possibilities may explain this observation. First, only Jurkat among many cancers displayed such a high susceptibility, suggesting that high sensitivity to FasL-mediated apoptosis is a property characteristic of the Jurkat target. A recent study has shown that Fas-mediated apoptosis in Jurkat cells triggers rapid release of ATP via pannexin1 channels, leading to an ATP-dependent purinergic receptor-mediated death [15]. Higher order structures formed due to aggregation of scFv component [16], so as to form scFv dimers/trimers of FasL_ext_ trimers could have enhance the cytotoxicity of FasL_ext_ moiety, as evidenced by higher cytotoxicity of both cc49 and L6 -scFv-FasL_ext_ on A20, HeLa and Jurkat cells compared to sFasL (Figure 3). Previous studies have shown that controlled cross-linking of sFasL trimers increased the cytotoxicity by over a 1000-fold as compared to uncross-linked sFasL, a property which is also applicable to soluble TRAIL and other members of the TNF super-family ligands [17]. Second, The unique multimeric structure of cc49scFv-FasL_ext_ (each Fas binding unit contains 3 TAG-72 binding sites) may allow more efficient binding to Jurkat TAG-72 Ag to outcompete anti-TAG-72 mAb. This will also add to the sensitivity of Jurkat to cc49scFv-FasL_ext_ cytotoxicity and resistance to cc83-mediated inhibition. Third, several TAG-72^+^ tumors increase their TAG-72 expression (10-100 folds) after implanting in athymic mice further leading to increased sensitivity of Jurkat-Ras tumors to cc49scFv-FasL_ext_*in vivo*[2]. Fourth, the great majority of TAG-72 has a molecular size of ≥10^6^. Although TAG-72 has been well established as the target Ag for cc49, high TAG-72 or TAG-72-related target sites that are reactive to cc49scFv-FasL_ext_ may be present on Jurkat since the reactive epitopes on Jurkat cells have not been characterized. These sites may be revealed or modified by the post-translational modifications of the TAG-72 [6].

Liver has been shown to be a target with lethal outcome when mice were treated with anti-Fas mAb Jo2 [2]. This lethal effect was originally attributed to the toxicity of hepatocytes. We demonstrated that FcRII knockout mice are resistant to Jo2 treatment [18]. In addition, anti-Fas mAb lacking FcRII binding activity also failed to induce lethality [18]. Moreover, sFasL was extremely ineffective in inducing lethality due to its inability to form aggregates on the target cells that lacked FcRII. Our study suggested that FcRII-expressing sinusoid endothelial cells in the liver are the real target for FcRII-dependent, Jo2-mediated lethality, which cannot be induced with scFv-FasL_ext_. The scFv-FasL_ext_ lacks FcRII binding activity and bears a ligand for tumor target Ag that selectively enhances binding to the cancer targets with minimal non-specific cytotoxicity against Fas-expressing normal cells that lack scFv targeted Ag.

There are potentially several ways to enhance the use of scFv-FasL_ext_ for therapy. First, any existing protocols for cancer therapies are likely to make the tumor target cells more sensitive toward the cytotoxicity of the scFv-FasL_ext_. Second, increase in Fas expression levels on target cells are often observed under various *in vivo* conditions. Amongst them, activation of cells and induction by cytokines such as IFN-γ almost always enhances Fas expression levels. Others have claimed that IFN-α or IFN-γ could induce higher expression of cancer Ag [19,20]. Our studies on many tumor cells cultured in the presence of IFN-γ only enhanced the cytotoxicity expression by 3-fold in a single *in vitro* treatment within 36 hours (pretreatment and cytotoxicity times), a level that may be helpful but not very effective. Additional studies are needed to determine if modulation of TAG-72 and TAL6 may be achieved to help the therapeutic effect of the cc49scFv-FasL_ext_ and L6scFv-FasL_ext_, respectively.

## Conclusions

Our studies demonstrate the reciprocal specificity of cc49scFv-FasL_ext_ and L6scFv-FasL_ext_ reagents and strongly suggest that targeted therapy of human cancers with FasL_ext_ based reagents is feasible, provided that the target antigen is expressed on the cancer cells and that the tumors are highly susceptible to FasL_ext_. Hematopoietic cancers are attractive targets due to their likelihood of FasL_ext_ sensitivity. The possibility of the use of interferons for modulation of tumor antigen expression and increase in susceptibility to L6scFv-FasL_ext_ -mediated cytotoxicity offers additional strategies. The eradication of Jurkat-Ras tumors with cc49scFv-FasL_ext_ without hepatic toxicity warrants the screening of human hematopoietic cancer repositories for TAG-72 and Fas expression and their subsequent curability with this reagent.

## Competing interests

The authors declare that they have no competing interests.

## Authors’ contribution

DVC derived the scFv-FasL_ext_ constructs, generated the overexpressing cell-lines and performed biochemical analyses. RS worked on epitope tagging, biochemical analyses, data presentation and wrote the manuscript. C-YAJ performed the in vivo experiments. SRR provided the scFv genes, cc83 and anti-L6 ascites and guidance with the use of these reagents. STJ provided concept development, financial support, conducted in vivo experiments and wrote the manuscript. All authors read and approved the final manuscript.

## Authors’ information

Derek V Chan in partial fulfillment of Ph.D thesis (2001-2004).
